# Syntheses and structures of two norpsilocin derivatives: *N*-ethyl-4-hy­droxy­tryptamine (4-HO-NET) and 4-hy­droxy-*N*-propyl­tryptamine (4-HO-NPT)

**DOI:** 10.1107/S2056989026008571

**Published:** 2026-08-25

**Authors:** Marilyn Naeem, Andrew R. Chadeayne, James A. Golen, David R. Manke

**Affiliations:** a University of Massachusetts Dartmouth 285 Old Westport Road North Dartmouth MA 02747 USA; b CaaMTech, Inc., 58 Sunset Way, Suite 209, Issaquah, WA 98027, USA; University of Aberdeen, United Kingdom

**Keywords:** crystal structure, tryptamines, indoles, hydrogen bonds

## Abstract

The solid-state structures of *N*-ethyl-4-hy­droxy­tryptamine (4-HO-NET) {systematic name: 3-[2-(ethyl­amino)­eth­yl]-1*H*-indol-4-ol}, C_12_H_16_N_2_O, and 4-hy­droxy-*N*-propyl­tryptamine (4-HO-NPT) {systematic name: 3-[2-(propyl­amino)­eth­yl]-1*H*-indol-4-ol}, C_13_H_18_N_2_O, are reported. Both compounds possess a single tryptamine mol­ecule in the asymmetric unit that exhibits an inter­nal O—H⋯N hydrogen bond. In the extended structures, the mol­ecules are linked by N—H⋯O hydrogen bonds to form infinite chains along [001] for 4-HO-NET and along [101] for 4-HO-NPT.

## Abstract 2

The solid state structures of two synthetic *N*-monoalkytryptamine analogs of the ‘magic’ mushroom natural product norpsilocin are presented.

## 1. Chemical context

Traditional psychoactive compounds including psilocybin (4-phosphor­yloxy-*N*,*N*-di­methyl­tryptamine; C_12_H_17_N_2_O_4_P), psilocin (4-hy­droxy-*N*,*N*-di­methyl­tryptamine; C_12_H_16_N_2_O), DMT (*N*,*N*-di­methyl­tryptamine; C_12_H_16_N_2_), and 5-MeO-DMT (5-meth­oxy-*N*,*N*-di­methyl­tryptamine; C_13_H_18_N_2_O) are all based upon an *N*,*N*-di­alkyl­tryptamine backbone. Lesser known naturally occurring compounds such as baeocystin (4-phosphor­yloxy-*N*-methyl­tryptamine; C_11_H_15_N_2_O_4_P) and norpsilocin (4-hy­droxy-*N*-methyl­tryptamine; C_11_H_14_N_2_O) have a suggested biological relevance but an underdeveloped chemistry. We have previously reported the crystal structures of both of these *N*-mono­alkyl­tryptamine compounds (Naeem *et al.*, 2022 ▸; Chadeayne *et al.*, 2020*b* ▸), as well as explored their pharmacological properties (Glatfelter, Pottie *et al.*, 2022 ▸). The removal of a single methyl group from the traditional *N*,*N*-di­methyl­tryptamines can significantly alter lipophilicity, central nervous system exposure, metabolism, receptor activity and transporter pharmacology. The *N*-mono­alkyl­tryptamines are a group of serotonergic chemicals that could present pharmacology not accessible through the class of *N*,*N*-di­alkyl­tryptamines. In early 2023, we reported the synthesis and structure of 4-hy­droxy-*N*-iso­propyl­tryptamine (4-HO-NiPT) as the first direct *N*-alkyl analogue of norpsilocin (Laban *et al.*, 2023 ▸). Sherwood and co-workers later reported a number of other *N*-alkyl analogues, exploring their pharmacology. Of note, derivatives of norpsilocin where the steric bulk of the alkyl group was increased (*i.e*. 4-HO-NiPT) showed psychoactive effects in animal models (Sherwood *et al.*, 2024 ▸). We recently reported the 4-meth­oxy variant of norpsilocin, as well as other 4-meth­oxy mono-*N*-alkyl derivatives, which were shown to be potent 5-HT_2A_ agonists with reduced psychedelic-like effects in animal models (Glatfelter, Schalk *et al.*, 2026 ▸). Herein, we report the syntheses and structures of two norpsilocin analogues, *N*-ethyl-4-hy­droxy­tryptamine (4-HO-NET; C_12_H_16_N_2_O) and 4-hy­droxy-*N*-prop­yl­tryptamine (4-HO-NPT; C_13_H_18_N_2_O).
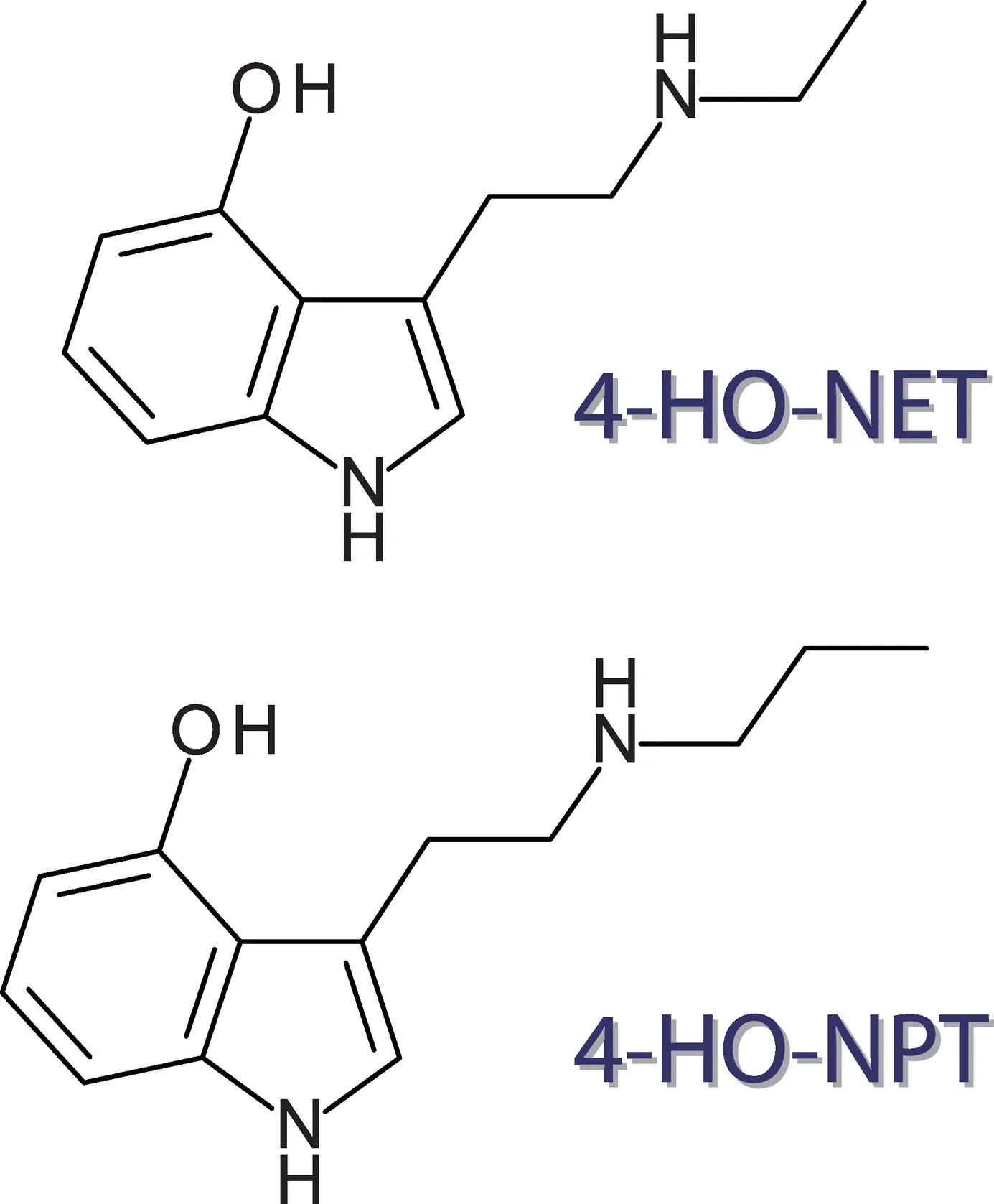


## 2. Structural commentary

**Figure 1:**
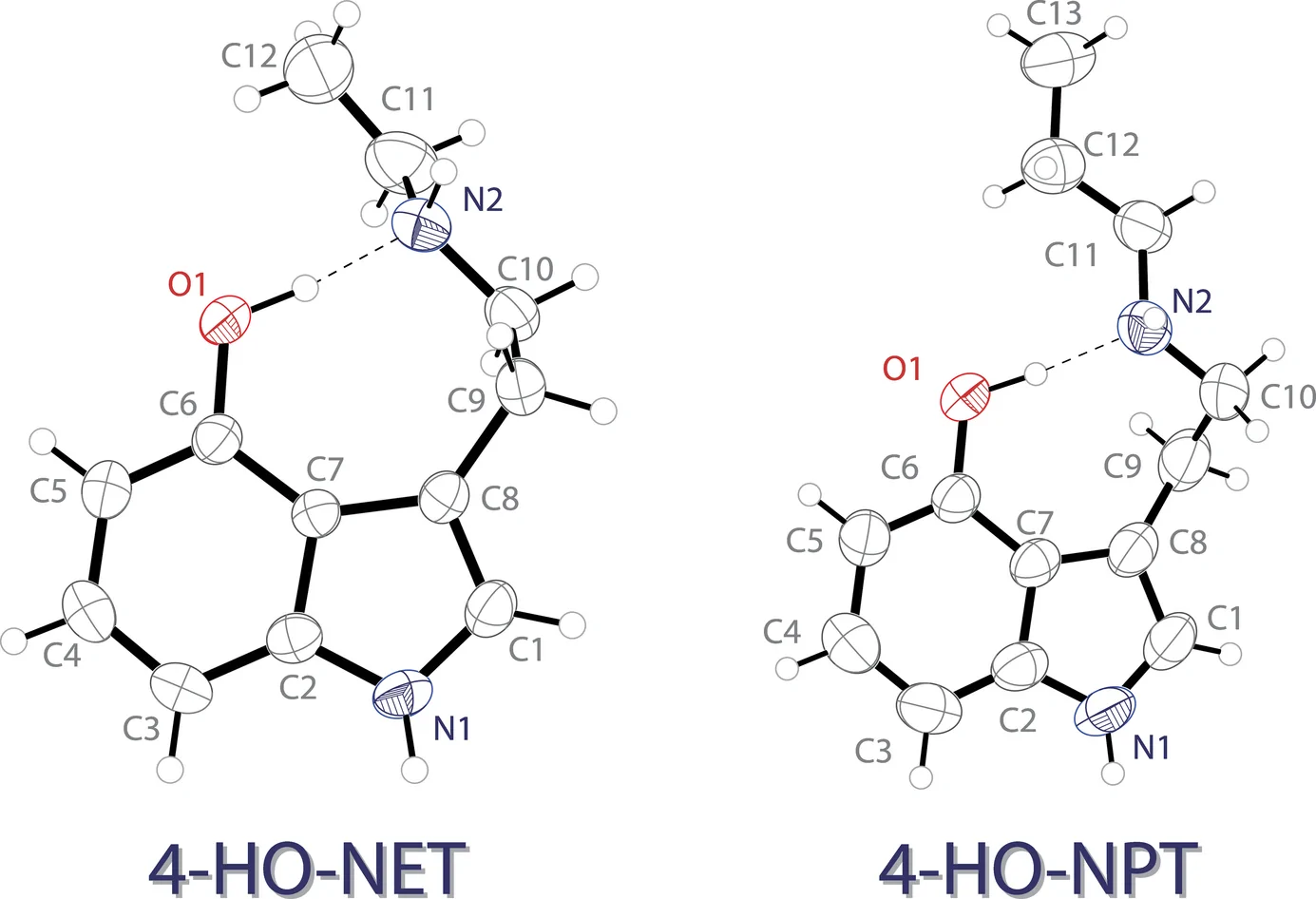
The mol­ecular structures of 4-HO-NET (left) and 4-HO-NPT (right) with displacement ellipsoids drawn at the 50% probability level. Hydrogen bonds are shown as dashed lines.

The mol­ecular structures of 4-HO-NET and 4-HO-NPT are shown in Fig. 1 ▸. The asymmetric unit of each structure contains a single tryptamine mol­ecule. The side chain in 4-HO-NET displays an extended conformation, as shown by the C9—C10—N2—C11 and C10—N2—C11—C12 torsion angles of −174.88 (12) and −174.34 (14)°, respectively. The longer side chain in 4-HO-NPT shows a more twisted conformation with C9—C10—N2—C11 = −101.41 (15), C10—N1—C11—C12 = 174.61 (13) and N2—C11—C12—C13 = 169.17 (13)°.

Both mol­ecules possess an inter­nal O—H⋯N hydrogen bond between the phenol oxygen atom (O1) and the acyclic amine nitro­gen atom (N2), which generates an *S*(8) ring in each case. As expected, both C1–C8/N1 indole ring systems are near planar, showing a r.m.s. deviation of 0.009Å for 4-HO-NET and 0.011Å for 4-HO-NPT.

## 3. Supra­molecular features

**Figure 2:**
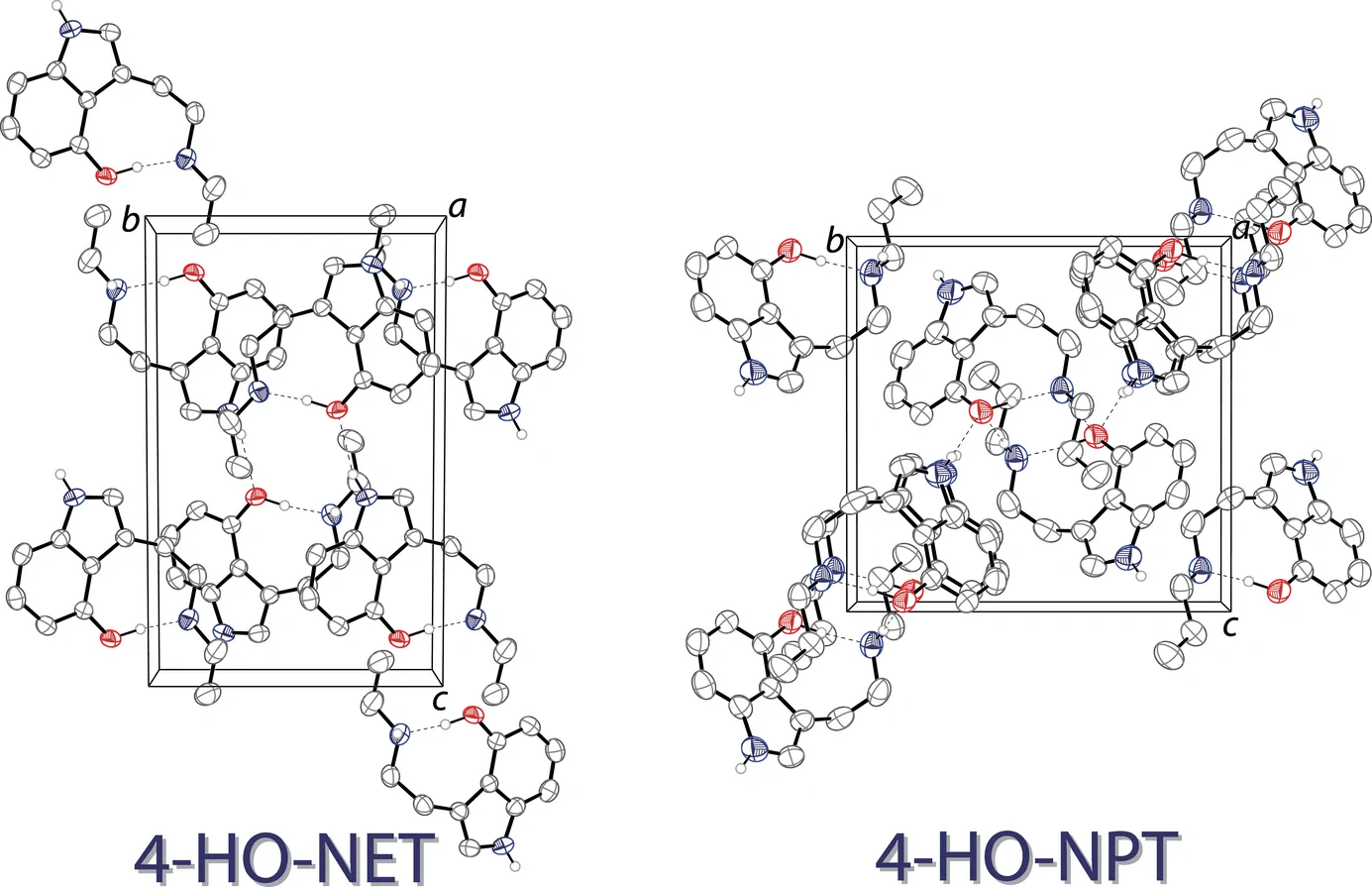
The crystal packing of 4-HO-NET (left) and 4-HO-NPT (right), both shown along the *a*-axis direction. Hydrogen bonds are shown as dashed lines. H atoms not involved in hydrogen bonding are omitted for clarity.

In the solid-state structure of 4-HO-NET, the mol­ecules are linked by N1—H1*A*⋯O1 hydrogen bonds between the indole N—H grouping and the hydroxide group. These hydrogen bonds link the mol­ecules into infinite chains propagating along the [001] direction (Table 1 ▸). The crystal packing of 4-HO-NET is shown on the left in Fig. 2 ▸.

In the extended structure of 4-HO-NPT, two mol­ecules are connected through N2—H2⋯O1 hydrogen bonds. These inter­molecular hydrogen bonds combine with the O1—H1⋯N2 intra­molecular hydrogen bonds to form rings demonstrating a graph set notation of 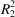
(16) (Etter *et al.*, 1990 ▸). These dimers are then joined together through the indole N1—H1A⋯O1 hydrogen bond to the next dimer, forming infinite chains propagating along the [101] direction (Table 2 ▸). The crystal packing of 4-HO-NPT is shown on the right in Fig. 2 ▸.

## 4. Database survey

The structures of mono­alkyl­tryptamines are not common in the Cambridge Structural Database, with only ten crystal structures previously reported, all in the past few years. These include the natural product baeocystin (Naeem *et al.*, 2022 ▸; FETBAB), its hydrolysis product norpsilocin as its freebase and fumarate salt (Chadeayne *et al.*, 2020*b* ▸; MULXAV, MULXEZ) and the synthetic prodrug of norpsilocin, 4-acet­oxy-*N*-methyl­tryptamine (Glatfelter, Pottie *et al.*, 2022 ▸). The others are *N*-methyl­serotonin as its hydrogen oxalate salt (Naeem, Anas *et al.*, 2023 ▸), 4-benzyl-*N*-iso­propyl­tryptamine, 4-hy­droxy-*N*-iso­propyl­tryptamine (Laban *et al.*, 2023 ▸3; YEYZIP, YEYZOV), and the freebase, bromide and fumarate salts of *N*-cyclo­hexyl­tryptamine (Naeem, Le *et al.*, 2023 ▸; YITWAD, YITWEH, YITWIL).

There are eight 4-hy­droxy-*N*,*N*-di­alkyl­tryptamine crystal structures reported in the literature, which are 4-hy­droxy-*N*-methyl-*N*-iso­propyl­tryptamine as its fumarate (Chadeayne *et al.*, 2020*a* ▸; TUFQAP) and hydro­fumarate (Chadeayne *et al.*, 2019*a* ▸; RONSUL), 4-hy­droxy-*N*,*N*-di­propyl­tryptamine as its fumarate (Chadeayne *et al.*, 2019*b* ▸; WUCGAF) and chloride (Sammeta *et al.*, 2020 ▸; WAMGEA) salts, 4-hy­droxy-*N*,*N*-diiso­propyl­tryptamine as its hydro­fumarate salt (Naeem *et al.*, 2025 ▸; BOWSOF), and the natural product psilocin as its freebase (Petcher & Weber, 1974 ▸; PSILIN; Zeller *et al.*, 2024 ▸; PSILIN03), its tatrate salt (Barrow *et al.*, 2025 ▸; XOWXIU), and as a co-crystal with the natural product psilocybin (Silverstone, 2024 ▸; MIMKOM). There are also four 4-hy­droxy-*N*,*N*,*N*-tri­alkyl­tryptamine crystal structures reported including 4-hy­droxy-*N*,*N*,*N*-tri­methyl­tryptamine (Chadeayne *et al.*, 2020*c* ▸; XUXFAA), 4-hy­droxy-*N*,*N*-dimethyl-*N*-ethyl­tryptamine, 4-hy­droxy-*N*,*N*-dimethyl-*N*-propyl­tryptamine, and 4-hy­droxy-*N*,*N*-dimethyl-*N*-iso­propyl­tryptamine (Glatfelter, Pham *et al.*, 2022 ▸; EDOYIJ, EDOYUV, EDOZEG).

## 5. Synthesis and crystallization

**4-HO-NET:** To an ice-bath-cooled diethyl ether (60 ml) solution of 4-benz­yloxy-1*H*-indole (3.0 g) was added oxalylchloride (3.4 g) dropwise. The mixture was stirred in the ice bath for 6 h and then added dropwise to an ice-bath-cooled solution of 70% aqueous ethyl­amine (5.8 g). The mixture was warmed to room temperature and stirred overnight. The resulting suspension was concentrated under reduced pressure and the residue was purified by silica gel chromatography (methyl­ene chloride/methanol) to afford 2-(4-benz­yloxy)-1*H*-indol-3-yl)-*N*-ethyl-2-oxoacetamide as a yellow oil (3.2 g). The yellow oil was dissolved in tetra­hydro­furan (50 ml), cooled in an ice bath, and 30 ml of 1 *M* borane-tetra­hydro­furan was added dropwise. The mixture was heated at reflux overnight. The resulting yellow solution was quenched with 2 *M* hydro­chloric acid and heated at reflux for 2 h. The mixture was cooled and ammonium hydroxide was added until the solution was basic. The resulting mixture was extracted with methyl­ene chloride; the organic layer was washed with water and brine, and then dried over sodium sulfate. Solvent was removed *in vacuo* and the resulting residue was purified by silica gel chromatography [methyl­ene chloride/methanol (3.*5 M* ammonia)] to afford 4-benz­yloxy-*N*-ethyl­tryptamine (4-BnO-NET) as a yellow solid (1.6 g, 54% yield). To a solution of 4-BnO-NET (600 mg) in methanol (12 ml) was added Pd/C (120 mg) and Pd(OH)_2_/C (120 mg). The mixture was stirred for 3 h under a hydrogen atmosphere. The resulting black suspension was filtered, the solid was washed with methanol, and the combined filtrates were concentrated under reduced pressure. The resulting residue was purified by silica gel chromatography [methyl­ene chloride/methanol (3.5 *M* ammonia)] to afford 4-hy­droxy-*N*-ethyl­tryptamine (4-HO-NET) as a white solid (162 mg, 39% yield). Single crystals suitable for X-ray diffraction were grown from the slow evaporation of an acetone solution.

**4-HO-NPT:** To an ice bath-cooled diethyl ether (40 ml) solution of 4-benz­yloxy-1*H*-indole (2.0 g) was added oxalylchloride (2.3 g) dropwise. The mixture was stirred for 6 h under cooling, and then added dropwise into an ice-bath cooled vessel containing propan-1-amine (5.3 g). The mixture was warmed to room temperature and stirred overnight. The solvent was removed *in vacuo* and the resulting residue was purified by silica gel chromatography (methyl­ene chloride/methanol) to afford 2-(4-benz­yloxy-1*H*-indol-3-yl)-2-oxo-*N*-propyl­acetamide as a yellow oil (1 g, 33% yield). This yellow oil was dissolved in tetra­hydro­furan (15 ml) and cooled in an ice bath, then 9 ml of 1.0 *M* borane-tetra­hydro­furan in tetra­hydro­furan was added dropwise. The mixture was heated at reflux for 2 h, cooled to room temperature, and then ammonium hydroxide was added until it was basic. The mixture was extracted with methyl­ene chloride and the organic phase was washed with water and brine. It was dried over sodium sulfate, then solvent was removed *in vacuo*, and purified by silica gel chromatography to yield 4-benz­yloxy-*N*-propyl­tryptamine (4-BnO-NPT) as a yellow solid (192 mg, 21% yield). To a solution of 4-BnO-NPT (190 mg) in methanol (4 ml) was added Pd/C (40 mg) and Pd(OH)_2_/C (40 mg). The mixture was stirred for 3 h under a hydrogen atmosphere. The resulting black suspension was filtered, the solid was washed with methanol, and the combined filtrates were concentrated under reduced pressure. The resulting residue was purified by silica gel chromatography [methyl­ene chloride/methanol (3.5 *M* ammonia)] to afford 4-hy­droxy-*N*-propyl­tryptamine (4-HO-NPT) as a white solid (75 mg, 55% yield). Single crystals suitable for X-ray diffraction studies were grown from the slow evaporation of an acetone solution.

## 6. Refinement

Crystal data, data collection and structure refinement details are summarized in Table 3 ▸. N- and O-bound H atoms were refined with restrained distances. C-bound H atoms were positioned geometrically (0.93–0.97 Å) and refined as riding with *U*_iso_(H) = 1.2–1.5*U*_eq_(C).

## Supplementary Material

Crystal structure: contains datablock(s) global, 4-HO-NPT, 4-HO-NET. DOI: 10.1107/S2056989026008571/hb8250sup1.cif

Structure factors: contains datablock(s) 4-HO-NET. DOI: 10.1107/S2056989026008571/hb82504-HO-NETsup4.hkl

Structure factors: contains datablock(s) 4-HO-NPT. DOI: 10.1107/S2056989026008571/hb82504-HO-NPTsup5.hkl

Supporting information file. DOI: 10.1107/S2056989026008571/hb82504-HO-NETsup4.cml

Supporting information file. DOI: 10.1107/S2056989026008571/hb82504-HO-NPTsup5.cml

CCDC references: 2581888, 2581887

Additional supporting information:  crystallographic information; 3D view; checkCIF report

## Figures and Tables

**Table 1:** Hydrogen-bond geometry (Å, °) for 4-HO-NET[Chem scheme1]

*D*—H⋯*A*	*D*—H	H⋯*A*	*D*⋯*A*	*D*—H⋯*A*
N1—H1*A*⋯O1^i^	0.87 (1)	2.06 (1)	2.8789 (15)	158 (2)
O1—H1⋯N2	1.00 (1)	1.58 (1)	2.5764 (16)	173 (2)

Symmetry code: (i) 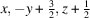.

**Table 2:** Hydrogen-bond geometry (Å, °) for 4-HO-NPT[Chem scheme1]

*D*—H⋯*A*	*D*—H	H⋯*A*	*D*⋯*A*	*D*—H⋯*A*
O1—H1⋯N2	1.01 (1)	1.59 (1)	2.5905 (15)	175 (2)
N1—H1*A*⋯O1^i^	0.87 (1)	2.06 (1)	2.9347 (16)	177 (2)
N2—H2⋯O1^ii^	0.90 (1)	2.62 (1)	3.4957 (17)	166 (2)

Symmetry codes: (i) 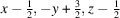; (ii) 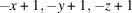.

**Table 3:** Experimental details

	4-HO-NET	4-HO-NPT
Crystal data		
Chemical formula	C_12_H_16_N_2_O	C_13_H_18_N_2_O
*M*_r_	204.27	218.29
Crystal system, space group	Monoclinic, *P*2_1_/*c*	Monoclinic, *P*2_1_/*n*
Temperature (K)	300	300
*a*, *b*, *c* (Å)	8.3887 (5), 9.0776 (5), 14.5657 (9)	9.0478 (6), 11.6680 (8), 11.3885 (7)
β (°)	100.132 (2)	91.523 (2)
*V* (Å^3^)	1091.87 (11)	1201.86 (14)
*Z*	4	4
*F*(000)	440	472
*D_x_* (Mg m^−3^)	1.243	1.206
Radiation type	Mo *K*α	Mo *K*α
No. of reflections for cell measurement	9957	8762
θ range (°) for cell measurement	2.7–26.3	2.8–26.4
μ (mm^−1^)	0.08	0.08
Crystal shape	Block	Block
Colour	Yellow	Colourless
Crystal size (mm)	0.27 × 0.21 × 0.20	0.24 × 0.22 × 0.21

Data collection		
Diffractometer	Bruker D8 Venture CMOS	Bruker D8 Venture CMOS
Scan method	φ and ω scans	φ and ω scans
Absorption correction	Multi-scan (*SADABS*; Krause *et al.*, 2015 ▸)	Multi-scan (*SADABS*; Krause *et al.*, 2015 ▸)
*T*_min_, *T*_max_	0.704, 0.745	0.705, 0.745
No. of measured, independent and observed [*I* > 2σ(*I*)] reflections	23801, 2243, 1953	34791, 2472, 1941
*R*_int_	0.028	0.040
(sin θ/λ)_max_ (Å^−1^)	0.625	0.626

Refinement		
*R*[*F*^2^ > 2σ(*F*^2^)], *wR*(*F*^2^), *S*	0.040, 0.112, 1.05	0.038, 0.107, 1.01
No. of reflections	2243	2472
No. of parameters	150	159
No. of restraints	3	3
H-atom treatment	H atoms treated by a mixture of independent and constrained refinement	H atoms treated by a mixture of independent and constrained refinement
Δρ_max_, Δρ_min_ (e Å^−3^)	0.25, −0.13	0.14, −0.11

Computer programs: *APEX3* and *SAINT* (Bruker, 2021 ▸), *SHELXT2014* (Sheldrick, 2015*a* ▸), *SHELXL2018* (Sheldrick, 2015*b* ▸), *OLEX2* (Dolomanov *et al.*, 2009 ▸) and *publCIF* (Westrip, 2010 ▸).

## supplementary crystallographic information

### 3-[2-(Ethylamino)ethyl]-1H-indol-4-ol (4-HO-NET) Crystal data

**Table:**

C_12_H_16_N_2_O	*F*(000) = 440
*M**_r_* = 204.27	*D*_x_ = 1.243 Mg m^−^^3^
Monoclinic, *P*2_1_/*c*	Mo *K*α radiation, λ = 0.71073 Å
*a* = 8.3887 (5) Å	Cell parameters from 9957 reflections
*b* = 9.0776 (5) Å	θ = 2.7–26.3°
*c* = 14.5657 (9) Å	µ = 0.08 mm^−^^1^
β = 100.132 (2)°	*T* = 300 K
*V* = 1091.87 (11) Å^3^	Block, yellow
*Z* = 4	0.27 × 0.21 × 0.20 mm

### 3-[2-(Ethylamino)ethyl]-1H-indol-4-ol (4-HO-NET) Data collection

**Table:**

Bruker D8 Venture CMOS diffractometer	1953 reflections with *I* > 2σ(*I*)
φ and ω scans	*R*_int_ = 0.028
Absorption correction: multi-scan (SADABS; Krause *et al.*, 2015)	θ_max_ = 26.4°, θ_min_ = 2.7°
*T*_min_ = 0.704, *T*_max_ = 0.745	*h* = −10→10
23801 measured reflections	*k* = −11→11
2243 independent reflections	*l* = −18→18

### 3-[2-(Ethylamino)ethyl]-1H-indol-4-ol (4-HO-NET) Refinement

**Table:**

Refinement on *F*^2^	Hydrogen site location: mixed
Least-squares matrix: full	H atoms treated by a mixture of independent and constrained refinement
*R*[*F*^2^ > 2σ(*F*^2^)] = 0.040	*w* = 1/[σ^2^(*F*_o_^2^) + (0.0518*P*)^2^ + 0.3161*P*] where *P* = (*F*_o_^2^ + 2*F*_c_^2^)/3
*wR*(*F*^2^) = 0.112	(Δ/σ)_max_ < 0.001
*S* = 1.05	Δρ_max_ = 0.25 e Å^−^^3^
2243 reflections	Δρ_min_ = −0.13 e Å^−^^3^
150 parameters	Extinction correction: SHELXL2018 (Sheldrick, 2015b), Fc^*^=kFc[1+0.001xFc^2^λ^3^/sin(2θ)]^-1/4^
3 restraints	Extinction coefficient: 0.007 (2)
Primary atom site location: dual	

### 3-[2-(Ethylamino)ethyl]-1H-indol-4-ol (4-HO-NET) Special details

**Table:**

Geometry. All esds (except the esd in the dihedral angle between two l.s. planes) are estimated using the full covariance matrix. The cell esds are taken into account individually in the estimation of esds in distances, angles and torsion angles; correlations between esds in cell parameters are only used when they are defined by crystal symmetry. An approximate (isotropic) treatment of cell esds is used for estimating esds involving l.s. planes.

### 3-[2-(Ethylamino)ethyl]-1H-indol-4-ol (4-HO-NET) Fractional atomic coordinates and isotropic or equivalent isotropic displacement parameters (Å^2^)

**Table:**

	*x*	*y*	*z*	*U*_iso_*/*U*_eq_	
O1	0.26670 (16)	0.64120 (12)	0.59539 (6)	0.0558 (3)	
N1	0.24109 (16)	0.75788 (14)	0.90599 (8)	0.0457 (3)	
N2	0.35265 (16)	0.37322 (13)	0.63603 (9)	0.0457 (3)	
C1	0.32861 (17)	0.63081 (16)	0.90504 (9)	0.0425 (3)	
H1B	0.373600	0.578230	0.958099	0.051*	
C2	0.19401 (15)	0.80509 (14)	0.81596 (9)	0.0365 (3)	
C3	0.10539 (17)	0.93092 (16)	0.78357 (10)	0.0444 (3)	
H3	0.068680	0.996395	0.824333	0.053*	
C4	0.07518 (17)	0.95333 (16)	0.68926 (11)	0.0462 (4)	
H4	0.017317	1.036282	0.665300	0.055*	
C5	0.12934 (17)	0.85438 (15)	0.62827 (10)	0.0439 (3)	
H5	0.105785	0.872798	0.564516	0.053*	
C6	0.21698 (16)	0.72971 (14)	0.65966 (9)	0.0362 (3)	
C7	0.25324 (14)	0.70272 (13)	0.75676 (8)	0.0313 (3)	
C8	0.34133 (15)	0.59139 (14)	0.81676 (9)	0.0354 (3)	
C9	0.43485 (16)	0.45785 (15)	0.79633 (10)	0.0422 (3)	
H9A	0.480105	0.411568	0.855178	0.051*	
H9B	0.524860	0.490499	0.767807	0.051*	
C10	0.34164 (18)	0.34175 (15)	0.73357 (10)	0.0451 (3)	
H10A	0.386353	0.245039	0.750729	0.054*	
H10B	0.229064	0.342004	0.741166	0.054*	
C11	0.2549 (2)	0.2752 (2)	0.56823 (13)	0.0634 (5)	
H11A	0.140925	0.290158	0.569964	0.076*	
H11B	0.281328	0.173480	0.584668	0.076*	
C12	0.2861 (3)	0.3053 (2)	0.47154 (13)	0.0805 (6)	
H12A	0.219642	0.241769	0.427983	0.121*	
H12B	0.398139	0.287178	0.469347	0.121*	
H12C	0.260572	0.406187	0.455488	0.121*	
H1A	0.229 (2)	0.8046 (19)	0.9562 (9)	0.067 (5)*	
H1	0.301 (2)	0.5388 (13)	0.6159 (14)	0.086 (6)*	
H2	0.4583 (12)	0.3621 (19)	0.6293 (12)	0.062 (5)*	

### 3-[2-(Ethylamino)ethyl]-1H-indol-4-ol (4-HO-NET) Atomic displacement parameters (Å^2^)

**Table:**

	*U*^11^	*U*^22^	*U*^33^	*U*^12^	*U*^13^	*U*^23^
O1	0.0953 (9)	0.0449 (6)	0.0303 (5)	0.0192 (6)	0.0194 (5)	0.0045 (4)
N1	0.0593 (7)	0.0476 (7)	0.0307 (6)	0.0011 (6)	0.0096 (5)	−0.0077 (5)
N2	0.0504 (7)	0.0407 (6)	0.0468 (7)	0.0037 (5)	0.0102 (5)	−0.0051 (5)
C1	0.0493 (8)	0.0449 (7)	0.0316 (6)	−0.0017 (6)	0.0026 (5)	0.0025 (5)
C2	0.0374 (6)	0.0373 (7)	0.0355 (6)	−0.0048 (5)	0.0084 (5)	−0.0039 (5)
C3	0.0433 (7)	0.0399 (7)	0.0518 (8)	0.0043 (6)	0.0126 (6)	−0.0084 (6)
C4	0.0419 (7)	0.0383 (7)	0.0576 (9)	0.0073 (6)	0.0067 (6)	0.0052 (6)
C5	0.0491 (8)	0.0437 (7)	0.0377 (7)	0.0039 (6)	0.0046 (6)	0.0064 (6)
C6	0.0426 (7)	0.0344 (6)	0.0324 (6)	−0.0008 (5)	0.0088 (5)	0.0010 (5)
C7	0.0319 (6)	0.0308 (6)	0.0320 (6)	−0.0038 (5)	0.0074 (5)	0.0000 (5)
C8	0.0374 (6)	0.0358 (6)	0.0331 (6)	−0.0028 (5)	0.0063 (5)	0.0021 (5)
C9	0.0438 (7)	0.0401 (7)	0.0424 (7)	0.0062 (6)	0.0065 (6)	0.0060 (6)
C10	0.0524 (8)	0.0341 (7)	0.0512 (8)	0.0051 (6)	0.0159 (6)	0.0025 (6)
C11	0.0675 (11)	0.0538 (9)	0.0665 (11)	0.0006 (8)	0.0049 (8)	−0.0181 (8)
C12	0.1077 (17)	0.0721 (13)	0.0539 (10)	0.0148 (12)	−0.0069 (10)	−0.0130 (9)

### 3-[2-(Ethylamino)ethyl]-1H-indol-4-ol (4-HO-NET) Geometric parameters (Å, º)

**Table:**

O1—C6	1.3539 (16)	C5—H5	0.9300
O1—H1	1.003 (9)	C5—C6	1.3827 (19)
N1—C1	1.3687 (19)	C6—C7	1.4147 (17)
N1—C2	1.3700 (17)	C7—C8	1.4506 (17)
N1—H1A	0.867 (9)	C8—C9	1.5018 (18)
N2—C10	1.4679 (19)	C9—H9A	0.9700
N2—C11	1.468 (2)	C9—H9B	0.9700
N2—H2	0.914 (9)	C9—C10	1.519 (2)
C1—H1B	0.9300	C10—H10A	0.9700
C1—C8	1.3569 (18)	C10—H10B	0.9700
C2—C3	1.3988 (19)	C11—H11A	0.9700
C2—C7	1.4159 (17)	C11—H11B	0.9700
C3—H3	0.9300	C11—C12	1.502 (3)
C3—C4	1.368 (2)	C12—H12A	0.9600
C4—H4	0.9300	C12—H12B	0.9600
C4—C5	1.394 (2)	C12—H12C	0.9600
			
C6—O1—H1	116.7 (12)	C1—C8—C7	105.70 (11)
C1—N1—C2	108.59 (11)	C1—C8—C9	122.11 (12)
C1—N1—H1A	124.3 (13)	C7—C8—C9	132.18 (11)
C2—N1—H1A	126.7 (13)	C8—C9—H9A	108.1
C10—N2—H2	108.0 (11)	C8—C9—H9B	108.1
C11—N2—C10	114.23 (13)	C8—C9—C10	116.60 (11)
C11—N2—H2	107.4 (11)	H9A—C9—H9B	107.3
N1—C1—H1B	124.3	C10—C9—H9A	108.1
C8—C1—N1	111.32 (12)	C10—C9—H9B	108.1
C8—C1—H1B	124.3	N2—C10—C9	109.52 (11)
N1—C2—C3	128.68 (12)	N2—C10—H10A	109.8
N1—C2—C7	107.74 (11)	N2—C10—H10B	109.8
C3—C2—C7	123.58 (12)	C9—C10—H10A	109.8
C2—C3—H3	121.5	C9—C10—H10B	109.8
C4—C3—C2	116.99 (12)	H10A—C10—H10B	108.2
C4—C3—H3	121.5	N2—C11—H11A	109.5
C3—C4—H4	119.3	N2—C11—H11B	109.5
C3—C4—C5	121.37 (13)	N2—C11—C12	110.58 (16)
C5—C4—H4	119.3	H11A—C11—H11B	108.1
C4—C5—H5	119.0	C12—C11—H11A	109.5
C6—C5—C4	122.04 (13)	C12—C11—H11B	109.5
C6—C5—H5	119.0	C11—C12—H12A	109.5
O1—C6—C5	117.88 (12)	C11—C12—H12B	109.5
O1—C6—C7	123.38 (11)	C11—C12—H12C	109.5
C5—C6—C7	118.72 (12)	H12A—C12—H12B	109.5
C2—C7—C8	106.65 (11)	H12A—C12—H12C	109.5
C6—C7—C2	117.29 (11)	H12B—C12—H12C	109.5
C6—C7—C8	136.06 (11)		
			
O1—C6—C7—C2	179.68 (12)	C3—C2—C7—C6	−1.21 (19)
O1—C6—C7—C8	0.0 (2)	C3—C2—C7—C8	178.54 (12)
N1—C1—C8—C7	−0.41 (15)	C3—C4—C5—C6	−0.5 (2)
N1—C1—C8—C9	178.50 (12)	C4—C5—C6—O1	−178.97 (13)
N1—C2—C3—C4	179.56 (14)	C4—C5—C6—C7	−0.4 (2)
N1—C2—C7—C6	179.46 (11)	C5—C6—C7—C2	1.16 (18)
N1—C2—C7—C8	−0.79 (14)	C5—C6—C7—C8	−178.49 (13)
C1—N1—C2—C3	−178.73 (14)	C6—C7—C8—C1	−179.60 (14)
C1—N1—C2—C7	0.55 (15)	C6—C7—C8—C9	1.7 (2)
C1—C8—C9—C10	121.65 (15)	C7—C2—C3—C4	0.4 (2)
C2—N1—C1—C8	−0.08 (16)	C7—C8—C9—C10	−59.77 (19)
C2—C3—C4—C5	0.5 (2)	C8—C9—C10—N2	90.62 (14)
C2—C7—C8—C1	0.73 (14)	C10—N2—C11—C12	−174.34 (14)
C2—C7—C8—C9	−178.02 (13)	C11—N2—C10—C9	−174.88 (12)

### 3-[2-(Ethylamino)ethyl]-1H-indol-4-ol (4-HO-NET) Hydrogen-bond geometry (Å, º)

**Table:**

*D*—H···*A*	*D*—H	H···*A*	*D*···*A*	*D*—H···*A*
N1—H1*A*···O1^i^	0.87 (1)	2.06 (1)	2.8789 (15)	158 (2)
O1—H1···N2	1.00 (1)	1.58 (1)	2.5764 (16)	173 (2)

Symmetry code: (i) *x*, −*y*+3/2, *z*+1/2.

### 3-[2-(Propylamino)ethyl]-1H-indol-4-ol (4-HO-NPT) Crystal data

**Table:**

C_13_H_18_N_2_O	*F*(000) = 472
*M**_r_* = 218.29	*D*_x_ = 1.206 Mg m^−^^3^
Monoclinic, *P*2_1_/*n*	Mo *K*α radiation, λ = 0.71073 Å
*a* = 9.0478 (6) Å	Cell parameters from 8762 reflections
*b* = 11.6680 (8) Å	θ = 2.8–26.4°
*c* = 11.3885 (7) Å	µ = 0.08 mm^−^^1^
β = 91.523 (2)°	*T* = 300 K
*V* = 1201.86 (14) Å^3^	Block, colourless
*Z* = 4	0.24 × 0.22 × 0.21 mm

### 3-[2-(Propylamino)ethyl]-1H-indol-4-ol (4-HO-NPT) Data collection

**Table:**

Bruker D8 Venture CMOS diffractometer	1941 reflections with *I* > 2σ(*I*)
φ and ω scans	*R*_int_ = 0.040
Absorption correction: multi-scan (SADABS; Krause *et al.*, 2015)	θ_max_ = 26.4°, θ_min_ = 2.8°
*T*_min_ = 0.705, *T*_max_ = 0.745	*h* = −11→11
34791 measured reflections	*k* = −14→14
2472 independent reflections	*l* = −14→14

### 3-[2-(Propylamino)ethyl]-1H-indol-4-ol (4-HO-NPT) Refinement

**Table:**

Refinement on *F*^2^	Hydrogen site location: mixed
Least-squares matrix: full	H atoms treated by a mixture of independent and constrained refinement
*R*[*F*^2^ > 2σ(*F*^2^)] = 0.038	*w* = 1/[σ^2^(*F*_o_^2^) + (0.0476*P*)^2^ + 0.2229*P*] where *P* = (*F*_o_^2^ + 2*F*_c_^2^)/3
*wR*(*F*^2^) = 0.107	(Δ/σ)_max_ < 0.001
*S* = 1.01	Δρ_max_ = 0.14 e Å^−^^3^
2472 reflections	Δρ_min_ = −0.11 e Å^−^^3^
159 parameters	Extinction correction: SHELXL2018 (Sheldrick, 2015b), Fc^*^=kFc[1+0.001xFc^2^λ^3^/sin(2θ)]^-1/4^
3 restraints	Extinction coefficient: 0.010 (2)
Primary atom site location: dual	

### 3-[2-(Propylamino)ethyl]-1H-indol-4-ol (4-HO-NPT) Special details

**Table:**

Geometry. All esds (except the esd in the dihedral angle between two l.s. planes) are estimated using the full covariance matrix. The cell esds are taken into account individually in the estimation of esds in distances, angles and torsion angles; correlations between esds in cell parameters are only used when they are defined by crystal symmetry. An approximate (isotropic) treatment of cell esds is used for estimating esds involving l.s. planes.

### 3-[2-(Propylamino)ethyl]-1H-indol-4-ol (4-HO-NPT) Fractional atomic coordinates and isotropic or equivalent isotropic displacement parameters (Å^2^)

**Table:**

	*x*	*y*	*z*	*U*_iso_*/*U*_eq_	
O1	0.67289 (11)	0.65319 (8)	0.46631 (9)	0.0625 (3)	
N1	0.37615 (16)	0.73704 (13)	0.13702 (12)	0.0744 (4)	
N2	0.65373 (13)	0.43935 (10)	0.40664 (11)	0.0581 (3)	
C1	0.4626 (2)	0.64513 (15)	0.11322 (13)	0.0727 (5)	
H1B	0.459562	0.605365	0.042428	0.087*	
C2	0.41003 (15)	0.77475 (12)	0.24805 (12)	0.0555 (4)	
C3	0.35237 (17)	0.86658 (14)	0.30944 (15)	0.0664 (4)	
H3	0.279287	0.913394	0.276289	0.080*	
C4	0.40760 (18)	0.88524 (13)	0.42067 (15)	0.0683 (4)	
H4	0.372585	0.946906	0.463557	0.082*	
C5	0.51543 (17)	0.81379 (12)	0.47138 (13)	0.0610 (4)	
H5	0.549839	0.828481	0.547572	0.073*	
C6	0.57192 (14)	0.72214 (11)	0.41115 (11)	0.0474 (3)	
C7	0.52103 (13)	0.70145 (11)	0.29519 (11)	0.0459 (3)	
C8	0.55428 (15)	0.61854 (12)	0.20607 (11)	0.0549 (3)	
C9	0.66469 (18)	0.52272 (14)	0.20690 (13)	0.0671 (4)	
H9A	0.759008	0.552462	0.235646	0.081*	
H9B	0.677518	0.497638	0.126609	0.081*	
C10	0.62556 (19)	0.41950 (13)	0.28017 (15)	0.0703 (4)	
H10A	0.521883	0.401214	0.266863	0.084*	
H10B	0.683237	0.354207	0.255279	0.084*	
C11	0.79192 (15)	0.38631 (13)	0.45205 (14)	0.0606 (4)	
H11A	0.873537	0.411109	0.404706	0.073*	
H11B	0.784125	0.303640	0.445430	0.073*	
C12	0.82328 (17)	0.41798 (14)	0.57805 (14)	0.0652 (4)	
H12A	0.733916	0.407145	0.621928	0.078*	
H12B	0.848746	0.498669	0.581958	0.078*	
C13	0.9464 (2)	0.34963 (17)	0.63607 (16)	0.0825 (5)	
H13A	0.960198	0.374269	0.716039	0.124*	
H13B	1.036130	0.361400	0.594605	0.124*	
H13C	0.921194	0.269711	0.634508	0.124*	
H1	0.664 (2)	0.5715 (10)	0.4388 (17)	0.109 (7)*	
H1A	0.3140 (18)	0.7706 (16)	0.0882 (14)	0.097 (6)*	
H2	0.5807 (16)	0.4070 (15)	0.4476 (15)	0.089 (6)*	

### 3-[2-(Propylamino)ethyl]-1H-indol-4-ol (4-HO-NPT) Atomic displacement parameters (Å^2^)

**Table:**

	*U*^11^	*U*^22^	*U*^33^	*U*^12^	*U*^13^	*U*^23^
O1	0.0740 (6)	0.0542 (6)	0.0577 (6)	0.0054 (5)	−0.0287 (5)	−0.0068 (4)
N1	0.0834 (9)	0.0706 (9)	0.0672 (8)	−0.0035 (7)	−0.0373 (7)	0.0081 (7)
N2	0.0528 (7)	0.0504 (7)	0.0708 (8)	0.0000 (5)	−0.0054 (6)	−0.0020 (6)
C1	0.0961 (12)	0.0687 (10)	0.0517 (8)	−0.0093 (9)	−0.0259 (8)	−0.0037 (7)
C2	0.0519 (7)	0.0557 (8)	0.0581 (8)	−0.0098 (6)	−0.0138 (6)	0.0105 (6)
C3	0.0568 (8)	0.0571 (9)	0.0849 (11)	0.0028 (7)	−0.0074 (7)	0.0147 (8)
C4	0.0762 (10)	0.0531 (8)	0.0759 (10)	0.0061 (7)	0.0057 (8)	−0.0005 (7)
C5	0.0782 (10)	0.0525 (8)	0.0519 (8)	−0.0015 (7)	−0.0044 (7)	−0.0035 (6)
C6	0.0495 (7)	0.0453 (7)	0.0469 (7)	−0.0073 (5)	−0.0082 (5)	0.0020 (5)
C7	0.0434 (6)	0.0478 (7)	0.0460 (7)	−0.0104 (5)	−0.0061 (5)	0.0038 (5)
C8	0.0598 (8)	0.0582 (8)	0.0462 (7)	−0.0103 (6)	−0.0092 (6)	−0.0018 (6)
C9	0.0679 (9)	0.0782 (11)	0.0548 (8)	0.0017 (8)	−0.0053 (7)	−0.0182 (7)
C10	0.0737 (10)	0.0549 (9)	0.0810 (11)	0.0047 (7)	−0.0234 (8)	−0.0175 (8)
C11	0.0556 (8)	0.0516 (8)	0.0741 (10)	0.0037 (6)	−0.0053 (7)	0.0035 (7)
C12	0.0639 (9)	0.0655 (9)	0.0661 (9)	−0.0032 (7)	−0.0010 (7)	0.0121 (7)
C13	0.0751 (11)	0.0896 (13)	0.0819 (11)	−0.0025 (9)	−0.0155 (9)	0.0224 (10)

### 3-[2-(Propylamino)ethyl]-1H-indol-4-ol (4-HO-NPT) Geometric parameters (Å, º)

**Table:**

O1—C6	1.3587 (15)	C6—C7	1.4078 (16)
O1—H1	1.006 (9)	C7—C8	1.4397 (19)
N1—C1	1.359 (2)	C8—C9	1.499 (2)
N1—C2	1.3659 (19)	C9—H9A	0.9700
N1—H1A	0.873 (9)	C9—H9B	0.9700
N2—C10	1.474 (2)	C9—C10	1.513 (2)
N2—C11	1.4762 (18)	C10—H10A	0.9700
N2—H2	0.902 (9)	C10—H10B	0.9700
C1—H1B	0.9300	C11—H11A	0.9700
C1—C8	1.3623 (19)	C11—H11B	0.9700
C2—C3	1.389 (2)	C11—C12	1.501 (2)
C2—C7	1.4142 (18)	C12—H12A	0.9700
C3—H3	0.9300	C12—H12B	0.9700
C3—C4	1.367 (2)	C12—C13	1.508 (2)
C4—H4	0.9300	C13—H13A	0.9600
C4—C5	1.397 (2)	C13—H13B	0.9600
C5—H5	0.9300	C13—H13C	0.9600
C5—C6	1.3763 (19)		
			
C6—O1—H1	111.6 (12)	C8—C9—H9A	108.4
C1—N1—C2	108.83 (12)	C8—C9—H9B	108.4
C1—N1—H1A	126.3 (13)	C8—C9—C10	115.57 (13)
C2—N1—H1A	124.7 (13)	H9A—C9—H9B	107.4
C10—N2—C11	113.53 (12)	C10—C9—H9A	108.4
C10—N2—H2	109.3 (12)	C10—C9—H9B	108.4
C11—N2—H2	105.6 (12)	N2—C10—C9	112.14 (12)
N1—C1—H1B	124.2	N2—C10—H10A	109.2
N1—C1—C8	111.52 (14)	N2—C10—H10B	109.2
C8—C1—H1B	124.2	C9—C10—H10A	109.2
N1—C2—C3	129.54 (13)	C9—C10—H10B	109.2
N1—C2—C7	107.23 (13)	H10A—C10—H10B	107.9
C3—C2—C7	123.23 (13)	N2—C11—H11A	109.3
C2—C3—H3	121.4	N2—C11—H11B	109.3
C4—C3—C2	117.17 (14)	N2—C11—C12	111.62 (13)
C4—C3—H3	121.4	H11A—C11—H11B	108.0
C3—C4—H4	119.3	C12—C11—H11A	109.3
C3—C4—C5	121.49 (15)	C12—C11—H11B	109.3
C5—C4—H4	119.3	C11—C12—H12A	108.7
C4—C5—H5	119.3	C11—C12—H12B	108.7
C6—C5—C4	121.49 (13)	C11—C12—C13	114.03 (14)
C6—C5—H5	119.3	H12A—C12—H12B	107.6
O1—C6—C5	118.93 (11)	C13—C12—H12A	108.7
O1—C6—C7	122.09 (12)	C13—C12—H12B	108.7
C5—C6—C7	118.96 (12)	C12—C13—H13A	109.5
C2—C7—C8	107.36 (11)	C12—C13—H13B	109.5
C6—C7—C2	117.62 (12)	C12—C13—H13C	109.5
C6—C7—C8	135.02 (12)	H13A—C13—H13B	109.5
C1—C8—C7	105.06 (13)	H13A—C13—H13C	109.5
C1—C8—C9	124.52 (14)	H13B—C13—H13C	109.5
C7—C8—C9	130.41 (12)		
			
O1—C6—C7—C2	176.68 (11)	C3—C2—C7—C6	1.52 (19)
O1—C6—C7—C8	−2.7 (2)	C3—C2—C7—C8	−178.94 (13)
N1—C1—C8—C7	0.13 (18)	C3—C4—C5—C6	0.7 (2)
N1—C1—C8—C9	−179.06 (14)	C4—C5—C6—O1	−177.74 (13)
N1—C2—C3—C4	−179.79 (15)	C4—C5—C6—C7	1.0 (2)
N1—C2—C7—C6	−178.60 (12)	C5—C6—C7—C2	−2.00 (18)
N1—C2—C7—C8	0.94 (15)	C5—C6—C7—C8	178.62 (14)
N2—C11—C12—C13	169.17 (13)	C6—C7—C8—C1	178.77 (15)
C1—N1—C2—C3	179.00 (15)	C6—C7—C8—C9	−2.1 (3)
C1—N1—C2—C7	−0.87 (17)	C7—C2—C3—C4	0.1 (2)
C1—C8—C9—C10	−107.09 (17)	C7—C8—C9—C10	73.94 (19)
C2—N1—C1—C8	0.5 (2)	C8—C9—C10—N2	−77.85 (16)
C2—C3—C4—C5	−1.2 (2)	C10—N2—C11—C12	174.61 (13)
C2—C7—C8—C1	−0.65 (15)	C11—N2—C10—C9	−101.41 (15)
C2—C7—C8—C9	178.47 (15)		

### 3-[2-(Propylamino)ethyl]-1H-indol-4-ol (4-HO-NPT) Hydrogen-bond geometry (Å, º)

**Table:**

*D*—H···*A*	*D*—H	H···*A*	*D*···*A*	*D*—H···*A*
O1—H1···N2	1.01 (1)	1.59 (1)	2.5905 (15)	175 (2)
N1—H1*A*···O1^i^	0.87 (1)	2.06 (1)	2.9347 (16)	177 (2)
N2—H2···O1^ii^	0.90 (1)	2.62 (1)	3.4957 (17)	166 (2)

Symmetry codes: (i) *x*−1/2, −*y*+3/2, *z*−1/2; (ii) −*x*+1, −*y*+1, −*z*+1.
